# Differences in cerebral structure among patients with amnestic mild cognitive impairment and patients with Alzheimer’s disease

**DOI:** 10.3389/fnagi.2024.1453051

**Published:** 2024-12-04

**Authors:** Xiaorui Cui, Mingpeng Li, Guanxiong Lei, Jie Wang, Jialin Pan, Sheng Zhu, Tao Wu, Liangyu Zou, Jianhui Yan

**Affiliations:** ^1^Department of Neurology, Affiliated Hospital of Xiangnan University, Chenzhou, China; ^2^Department of Cardiovascular, Chenzhou No.1 People’s Hospital, The First Affiliated Hospital of Xiangnan University, Chenzhou, China; ^3^Department of Otolaryngology Head and Neck Surgery, Affiliated Hospital of Xiangnan University, Chenzhou, China; ^4^Clinical Medical Technology Demonstration Base For Auditory Disease In Hunan Province, Key Laboratory of Medical Imaging and Artificial Intelligence of Hunan Province, Hunan Engineering Research Center of Advanced Embedded Computing and Intelligent Medical Systems, Xiangnan University, Chenzhou, China; ^5^International Medical Center (Department of Geriatric Medicine), Shenzhen University General Hospital, Shenzhen, China; ^6^Department of Internal Medicine, Second People’s Hospital, Shenzhen, China; ^7^Department of Nuclear Medicin, Affiliated Hospital of Xiangnan University, Chenzhou, China; ^8^Department of Radiology, Affiliated Hospital of Xiangnan University, Chenzhou, China; ^9^Department of Neurology, Shenzhen People’s Hospital (First Affiliated Hospital of Southern University of Science and Technology), Second Clinical College, Jinan University, Shenzhen, China; ^10^Department of Neurology, Affiliated Hospital of Xiangnan University, Chenzhou, China

**Keywords:** Alzheimer’s disease, mild cognitive impairment, SBM, VBM, sMRI

## Abstract

**Background:**

Brain has been shown to undergo progressive atrophy in patients with Alzheimer’s disease (AD); however, more evidence is needed to elucidate how the brain structure changes during the progression to AD. Here, we observed differences in the cerebral structure among patients with amnestic mild cognitive impairment (aMCI) and patients with AD.

**Methods:**

A total of 46 participants were selected and divided into AD, aMCI, and healthy control (HC) groups. Structural magnetic resonance imaging (sMRI) was performed on all participants. Voxel-based morphometry (VBM) and surface-based morphometry (SBM) techniques were utilized to analyze sMRI data so as to identify significant differences among the specific brain regions of these three groups. Then, a correlation analysis was performed on the characteristics of the identified brain regions and the Mini-Mental State Examination (MMSE) and Montreal Cognitive Assessment (MoCA) cognitive assessment scores.

**Results:**

The volume of the left precuneus region, which was identified by voxel-based morphometry, and the thickness of both sides of the inferior parietal, which was identified by surface-based morphometry, were shown to be less in AD/aMCI patients, compared to those of the HC. The correlation analysis showed that there were significant differences between the volume of the left precuneus region and the MMSE/MoCA scores, as well as between the thickness of the left and right sides of the inferior parietal region and the MMSE/MoCA scores.

**Conclusion:**

The sMRI characteristics of the identified brain regions were considered to be potential predictive diagnostic biomarkers for AD.

**Systematic review registration:**

Identifier: ChiCTR2400092593.

## Introduction

Dementia in the elderly significantly impacts their physical and mental well-being, emerging as a primary global concern due to its social and economic implications (Dugu et al., 2003; Rosenblatt, 2005; Feczko, 2014; Kowalska et al., 2019). Alzheimer’s disease (AD), identified as the most prevalent cause of dementia, is a neurodegenerative condition with a latent onset and gradual progression, primarily characterized by consistent cognitive decline (Breijyeh and Karaman, 2020; Cooray et al., 2020; Zhang et al., 2021). According to World Health Organization, the number of AD patients worldwide is over 30 million and will be over 100 million by 2050, AD will become a major challenge for public health and increase the burden on society (van Oostveen and de Lange, 2021; Giridharan et al., 2022; Liu et al., 2022). Pathologically, AD exhibits the senile plaque containing β amyloid protein (Aβ), the neurofibrillary tangle with hyperphosphorylated tau protein, and degeneration and loss of acetylcholinergic neurons (Rajmohan and Reddy, 2017; Marcucci and Kleiman, 2021; Li and Gu, 2023). These pathological changes first occurred in the hippocampus and cerebral cortex. The onset of AD is insidious and progresses gradually, and only patients showing cognitive function impairment or mental state decline can be diagnosed clinically. Symptoms of these conditions include memory, attention, language and emotional disorders, and mental behavior abnormalities. Presently, there are limited therapeutic strategies available for the treatment of AD. They may alleviate some AD symptoms, but cannot reverse the course of the disease. Therefore, accurate diagnosis at the early stage of AD has great therapeutic potential (Regan et al., 2019; de Oliveira et al., 2020; Mahaman et al., 2022). However, due to the asymptomatic nature of AD progression, an accurate diagnosis cannot be determined until the patient exhibits severe cognitive dysfunction or mental state deterioration, thus missing the optimum treatment window and ultimately leading to death. Therefore, it is crucial to make a precise diagnosis of AD in its early stages so that AD progression can be effectively delayed.

Amnestic mild cognitive impairment (aMCI), which is featured as forgetting conversations and misplacing items, is memory-specific and considered to be the early stage of dementia (Sundermann et al., 2021; Tomoto et al., 2021; De Wit et al., 2022). According to reports, those with aMCI have a chance of developing AD that is at least five times more severe than that of healthy senior citizens (Jagust, 2008; Whitwell et al., 2008; Balthazar et al., 2010). However, timely detection of aMCI, proper physical activity, and adequate dietary supplementation were thought to be the most effective ways to halt the progression of aMCI to AD. Previous research revealed a strong link between the concentration of Aβ/tau in cerebrospinal fluid (CSF) and cognitive impairment, suggesting that tau may serve as a viable biomarker for the diagnosis of AD (Jia et al., 2019; Wang et al., 2020). Jack Jr. et al. proposed CSF Aβ42, Aβ42/Ab40 ratio, or CSF phosphorylated tau as the biomarker for AD diagnosis (Jack et al., 2018). However, the invasive examination cannot be used generally. More efficient, non-invasive, and practical diagnostic tests are required for assessing cognitive impairment.

Structural magnetic resonance imaging (sMRI) is a method of using magnetic resonance imaging (MRI) to obtain information about the internal structure of the human body. It can also specifically show each individual brain’s anatomical structure (Feng et al., 2022; Zhu et al., 2023). sMRI has been used to determine the stage and progression of AD. In patients with simple MCI or AD, sMRI was strongly associated with measures of cognitive function, and it showed more prediction accuracy in the progression of aMCI to AD than CSF tau, which were considered to be the biomarker for AD diagnosis for a long time (Brier et al., 2016). In 2018, National Institute on Aging and Alzheimer’s Association proposed a research framework on AD diagnosis with biomarkers in living persons, and included imaging results as one of different biomarkers based on the pathological process. This system was flexible and any biomarker that was available can be added (Jack et al., 2018). Financial capacity in Greek aMCI patients strongly correlates with right amygdala and left angular gyrus volumes, suggesting that emotion as well arithmetic skills are involved in financial capacity (Giannouli and Tsolaki, 2019). MRI data indicated that hippocampal volume, fractional anisotropy in cingulum and fornix, and functional connectivity within the default-mode network showed consistent associations with cognitive performance in all types of acute onset brain injury (Verhulst et al., 2023). The parahippocampal gyrus and hippocampus showed significant positive correlations with the severity of cognitive impairment as shown by MRI measurement (Zhao et al., 2023). Voxel-based morphometry (VBM) and surface-based morphometry (SBM), techniques for sMRI analysis, can be used to quantify the changes that have occurred in a number of sub-cortical regions of brain, including the hippocampus and the amygdaloid nucleus. The VBM and SBM analyses in sMRI demonstrated excellent potential for investigating imaging biomarkers for early-stage AD diagnosis (Cui et al., 2019; Thomann et al., 2021). The cortical volume, area and thickness (SBM) and left/right hippocampus and left amygdala (VBM) were found to be significantly changed in AD patients (Rechberger et al., 2022). Thus, more investigation should be performed on MCI and AD by using MRI with VBM and SBM analyses, in order to obtain more data and evidence for MCI and AD diagnosis and therapy.

In this study, under the hypothesis that brain structure changes in MCI and AD patients and monitoring the changes may help the diagnose and cure of dementia in clinic, we explored the structural changes of the brain in both aMCI and AD patients with MRI. We collect the basic information, neurological evaluation reports, and sMRI data. The VBM and SBM analyses were used to identify the exact brain regions that might serve as biomarkers for the diagnosis of aMCI and AD. We found that the volume of the left precuneus region and the thickness of the left/right inferior parietal showed decreased in aMCI and AD patients, compared to those of healthy control. This study may provide more data and evidence for MRI features in aMCI and AD, and the results might provide the basis and direction for clinical diagnosis and treatment of MCI and AD.

## Materials and methods

### Participants

This study was approved by Medical Ethics Committee of Shenzhen People’s Hospital (No. LL-KY-2019610). A total of 46 patients, who were admitted to the neurology department of Shenzhen People’s Hospital due to cognitive decline from January 2021 to December 2022, were selected. Combined with laboratory examination and quality control of image data, 20 cases were excluded, and a total of 26 patients (10 AD patients and 16 aMCI) were enrolled. In the same period, 12 patients with normal cognitive function were selected as control group. The diagnosis was performed by two qualified clinical neurologists who were completely blind to our study. The National Institute on Aging and Alzheimer’s Association (NIA-AA) guideline, which was released in 2011, served as the criteria for the diagnosis of AD. The *Diagnostic and Statistical Manual of Mental Disorders, Fifth Edition* (DSM-5) served as the criteria for the diagnosis of aMCI: (1) subjectively perceived memory loss; (2) objective assessment on MCI evidence; (3) impaired ability to live and social function; (4) Hachinski Ischemia Scale (HIS) ≤4 points, excluding cognitive decline caused by other specific causes; (5) duration of disease >3 months; (6) did not meet the diagnostic criteria for dementia.

Twelve sex-matched healthy controls were selected and met the following inclusion requirements: (1) no history of cognitive impairment; (2) perfect scores on the activities of daily living (ADL); (3) no relatives in the first degree who have AD: (4) no history of cerebrovascular illness. Written informed consent was obtained for all the participants, and this study was approved by the ethics committees of Shenzhen People’s Hospital.

### Laboratory examination

Every participant underwent the following standard examinations: regular blood test, basic liver and kidney function, thyroid and parathyroid function, electrolytes, blood sugar, HIV, *Treponema pallidum* antibody, homocysteine, sedimentation of the erythrocytes rate, folate, vitamin B12, heavy metals, tumor indicators, paratumor antibodies, drug or toxin tests, basic immune testing and other conditions affecting the metabolism and endocrine systems.

### Neuropsychological evaluations

The following routine cognitive scale screening was completed: Mini-Mental State Examination (MMSE), Montreal Cognitive Assessment (MoCA), ADL, and Hachinski Ischemic Score (HIS; Finney et al., 2016). They were also used for grouping of patients (AD or aMCI). In MMSE, illiteracy participants having MMSE score of ≤17 points, those with primary school education level having an MMSE score of ≤20 points, and those with middle school or above education level having an MMSE score of ≤24 points were classified as cognitive dysfunction. In the MoCA, the MoCA score ≥ 26 is considered to be normal cognitive function, and participants with an education of ≤12 years have one point more on the basic evaluation score (Seijo-Martinez et al., 2016). In ADL, 0–35 points are basically complete assistance; 35–80 points are partial assistance in wheelchair life; 80 points are wheelchair self-care level; 80–100 points are mostly self-care in ADL; and 100 points are complete self-care in ADL (Mlinac and Feng, 2016). HIS scale was used to exclude vascular cognitive dysfunction: HIS≤4 points, AD-derived cognitive dysfunction; 4 < HIS≤7, mixed dementia; HIS>7 points, highly indicative of vascular cognitive dysfunction. Only those with HIS≤4 points can be included in the patient group.

### MRI data acquisition

sMRI data of all participants were obtained using a 3.0 T scanner (Lumina, 3D mprage, Siemens) at Shenzhen People’s Hospital. The T1-weighted images were acquired using a brain volume sequence with the following parameters: TR 1900 ms, TE 2.26 ms, TI 900 ms, FA 9°, matrix 256 × 256, FOV 25.6 cm × 25. 6 cm, thickness = 1 mm, sagittal slice = 192, vovel size = 1 × 1 × 1 mm^3^.

### VBM analysis

VBM was performed in the SPM12 software package of MATLAB (2021b) platform (http://www.fil.ion.ucl.ac.uk/spm/; Chen et al., 2021). All T1-weighted images were first evaluated for image quality, and qualified ones were included in further study, which were spatially normalized in the standardized space using the template provided by the Montreal Neurological Institute (MNI). Each structural image was segmented into gray matter, white matter, and cerebrospinal fluid using a fully automated algorithm within SPM12 and subsequently transformed to the MNI space using diffeomorphic anatomical registration through exponentiated Lie algebra normalization. Next, the normalized gray matter images were smoothed (FWHM = 8 mm) for statistical analyses. During this procedure, comparisons between groups were performed between brain regions that showed significant differences in the ANOVA. The age and sex of each participant were entered into the design matrix as nuisance variables. The global volumes in the voxel intensities were used as confounding covariates. Voxel-wise false discovery rate (FDR) correction was used for multicomparison correction to control type I error (*p* < 0.01, FDR correction, minimum cluster size >100 voxels). The significance threshold of voxel level was <0.01 and the significance threshold of the cluster level was each considered to be <0.05. The anatomical position of each cluster via T (or F) test images was performed using Xjview software.^1^

### SBM analysis

Data analysis was performed using FreeSurfer (v6.0.0; https://surfer.nmr.mgh.harvard.edu/; Chen et al., 2021), which has been more used in the automatic analysis of neuroimaging data (Biffen et al., 2020; Brown et al., 2020). The software automatically distinguishes the cortical structure, and the original data of each participant needs to be pre-processed for about 20 h before the formal brain imaging data analysis. The processing steps include image reconstruction, correction of brain tissue, conversion of T1 images to the Talairach template to obtain accurate coordinates, image brightness correction, 3D brain image reconstruction, cortical surface expansion, mapping of spherical brain image sets, etc.

Five indexes, including thickness, volume, surface area, sulc (sulcus depth), and curv (curvature) were calculated based on FreeSurfer preprocessed data (Caunca et al., 2020). The calculated index was smoothed, and the smoothing core was 10 mm. Then, these five indexes were statistically analyzed, and gender and age were included in the covariates. The Different Onset and Different Slope model was selected as the statistical model. In adjusting for Monte Carlo multiple comparison correction, *p* < 0.001 for the vertex level and *p* < 0.05 for the lump level were considered to be significant. The values of the brain regions with significant differences were extracted, and the two-pair *t* test was used for *post hoc* analysis.

### Statistical analysis

A descriptive analysis of demographical variables, including age, sex, and clinical measures was performed. The Chi-squared or *t* test was used to analyze significant differences among the three groups, and *p* < 0.05 was considered to be statistically significant. For SBM/VBM data, analysis of variance (ANOVA) was performed among HC, aMCI and AD groups, and *p* < 0.05 was considered to be statistically significant. The differences between the groups were further explored by *post hoc* analysis. The values of brain regions with significant differences were extracted and analyzed by two-sample *t* test. By using MATLAB, the Pearson correlation analysis between the values of the significantly different brain areas obtained from SBM/VBM and MMSE/MoCA scores was performed, with the age and gender of individuals used as concomitant variables.

## Results

### Demographic and cognitive characteristics

Demographic characteristics, including age and sex, are shown in Table 1. The age was significantly different among the AD, aMCI, and HC groups, while gender distribution was not. As expected, neuropsychological scales (MMSE/MoCA) showed marked differences, with the performance in AD patients being worst, followed by that of aMCI patients, in comparison with that of HC patients.

**Table 1:** Demographic information.

Characteristic	AD, *N* = 10^1^	aMCI, *N* = 16^1^	HC, *N* = 12^1^	χ^2^/*t*	*p* value^2^
Age (years)	67.50 (7.50)	63.88 (7.96)	53.58 (12.35)	8.1	0.017
Gender (male/female)	3/10 (30%)	6/16 (38%)	3/12 (25%)		>0.9
MMSE	18.30 (2.63)	26.56 (2.71)	30.00 (0.00)	31	<0.001
MoCA	11.50 (3.10)	22.38 (3.84)	30.00 (0.00)	33	<0.001

1 Normally distributed data are described as the mean [standard deviation; M (SD)], and non-normally distributed data are described as the median (interquartile range) [M/(IQR)].

2 Kruskal–Wallis rank sum test; Fisher’s exact test.

### Brain volume changes and correlation results

**Figure 1:**
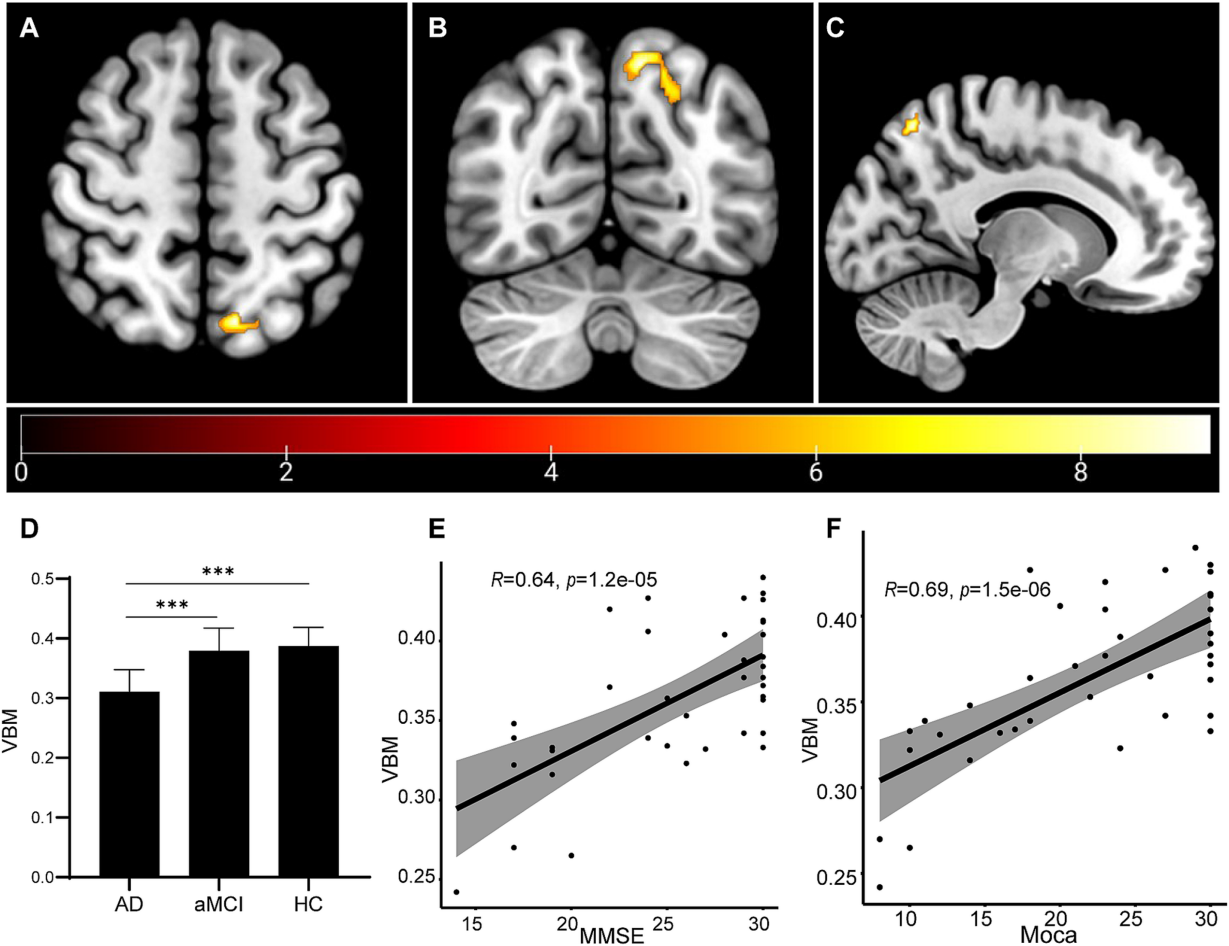
Brain volume changes as measured by VBM analysis. **(A–C)** The VBM analysis identified that the left Precuneus region showed atrophy in the AD group. The statistical result was shown **(D)**. Correlation analyses were performed between the volume of the left Precuneus region and MMSE score **(E)** and between the volume of the left precuneus region and MoCA score **(F)**. ***p < 0.001.

VBM was used to determine whether the gray matter (volume index) of the whole brain has atrophy and the atrophy area. For the VBM data, ANOVA of the three groups was performed, and then *t* test between the two groups is conducted after positive results are found. Our results showed that the left precuneus region was the peak MNI coordinate region, with a peak intensity of 9.2173 and a cluster size of 406 voxels in the AD group (Figures 1A–C). In addition, when compared to the aMCI and HC participants, the AD patients presented much less volumes in the left precuneus region (aMCI vs. AD: *p* = 1.26e-4; HC vs. AD: *p* = 4.08e-5). However, no significant difference was shown between the aMCI group and the HC group (*p* > 0.05; Figure 1D). By correlation analysis, the volume of the left precuneus region displayed a significant correlation with either the MMSE score or MoCA score (Figures 1E,F). These data implied that the left precuneus region may be involved in the process of the impairment of cognitive functions in AD.

### Thickness change of the cortex and correlation analysis

**Figure 2:**
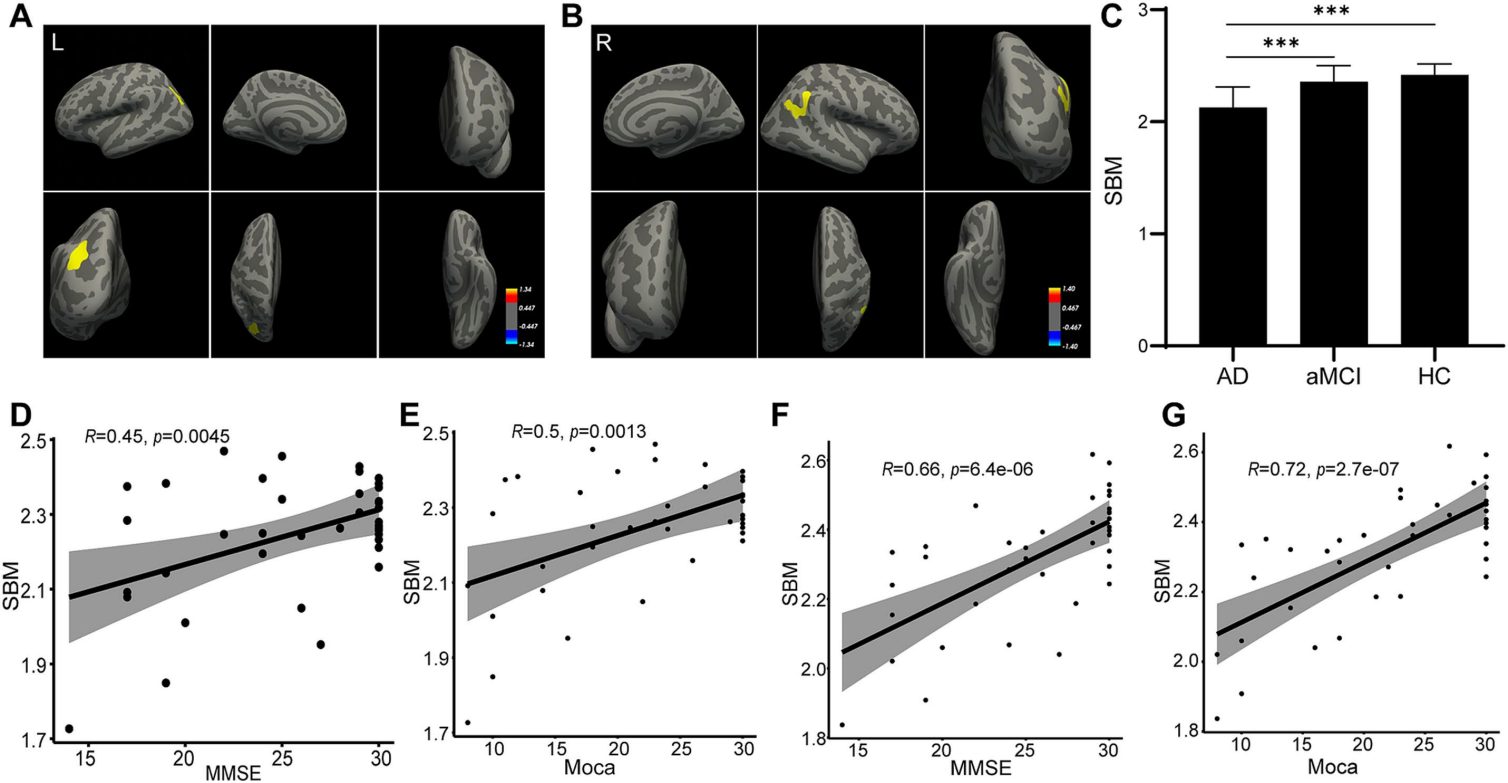
Thickness change as measured by SBM analysis. The SBM analysis identified that the thickness of the left **(A)** and right **(B)** inferior parietal regions changed in the AD group. The statistical result is shown **(C)**. Correlation analyses were performed between the thickness of the left inferior parietal region and MMSE score **(D)**, between the thickness of the left inferior parietal region and MoCA score **(E)**, between the thickness of the right inferior parietal region and MMSE score **(F)**, and between the thickness of right inferior parietal region and MoCA score **(G)**.

For the results of SBM analysis, it was divided into several indexes: thickness, volume, area, sulc and curv. First, ANOVA analysis was performed for the three groups, and then *t* test is conducted between the two groups after positive results are obtained. Our SBM analysis showed that, compared with the HC and aMCI participants, the thickness of the left inferior parietal region showed atrophy (Figure 2A). Meanwhile, the right inferior parietal also atrophied more sufficiently in the AD group than in the other two groups (Figure 2B). In addition, when compared to the aMCI and HC groups, the AD participants presented thinner in the left/right inferior parietal region (*p* < 0.001). However, no significant difference was shown between the aMCI group and the HC group (*p* > 0.05; Figure 2C). By correlation analysis, the thickness of either left or right inferior parietal region displayed a significant correlation with either MMSE score or MoCA score (Figures 2D–G). The SBM results indicated that both the left and right inferior parietal may contribute to the pathogenesis of AD.

## Discussion

sMRI, a non-invasive imaging technique, has been widely used in measuring the brain’s anatomical structure clinically. It has been used in the quantification of the sub-cortical region of the brain in many diseases, such as AD, with the use of VBM and SBM analyses. In this study, we used VBM and SBM to evaluate sMRI data from the AD, aMCI, and HC participants and found that the left precuneus and inferior parietal on both sides may play a role in the pathogenesis of AD. There was a statistically significant difference in the correlation study between the brain region and the MMSE or MoCA scores. Our research might suggest a possible biomarker as the sign from aMCI to AD.

There have been studies previously in MRI biomarker for MCI due to AD, but no conclusive results were achieved until now. Brain atrophy, especially hippocampus atrophy, was used as a diagnostic feature for AD. Atrophy in other brain regions, such as frontal and parietal regions, cortical thinning, and alternation in white matter were all correlated with memory deficits in AD early stage. Besides, brain lob hyperactivation/hypoactivation were also shown in early AD (Bayram et al., 2018). Khatri and Kwon developed a framework, which combined the hippocampal subfield, the amygdala volume, and brain networks with multiple measures of sMRI could improve AD diagnosis (Khatri and Kwon, 2022). Here in the present study, the volume of left precuneus and the thickness of inferior parietal on both sides may serve as aMCI due to AD biomarkers. Considering all previous and present studies on the MRI biomarkers for aMCI to AD, it’s urgent to conduct a comparative and large-population based study to reach a conclusion on the MRI biomarker. Furthermore, the intrinsic mechanism in the MRI feature changes and AD pathological alternation (such as tau and Abeta) were also needed to be investigated.

We investigated whether the left precuneus region may be engaged in the progression to AD based on VBM on all sMRI data from patients, and the results showed the AD patients presented much less volumes in the left precuneus region, compared to the aMCI group and the HC group, and the volume of the left precuneus region displayed a significant correlation with either the MMSE score or MoCA score (Figure 1). With the cognitive ability decreased, the volume of left precuneus region decreased. This suggest the left precuneus region volume might correlate with the cognitive ability. Prior research suggested that the precuneus was a crucial region for the memory deficit seen in early AD, which was probably caused by the disconnection mechanisms. According to Marco et al., high-frequency repeated transcranial magnetic stimulation on the precuneus was a successful treatment strategy for addressing memory loss in patients with early-stage AD (Koch et al., 2018). Giannouli et al. reported the correlation of amygdala volumes and metacognitive deficits in aMCI patients (Giannouli and Tsolaki, 2023). Additionally, Maria-Joao et al. identified precuneus metabolism in AD, MCI, and HC, and discovered that the precuneus region had the lowest glucose uptake efficiency, indicating that precuneus hypometabolism occurred more so in AD compared to MCI and HC. The conclusion implied that the pathophysiologic shift might serve as a biomarker for AD (Bailly et al., 2015). According to the results of our investigation, the precuneus was the greatest MNI coordinate location. Combined with above research results, it was possible that the precuneus could be used as a diagnostic biomarker. A number of other papers that did mention mechanisms might be useful references. According to Koen et al., several CSF biomarkers, such as visinin-like protein 1, neurogranin, BACE1, and Aβ1-40, were all correlated negatively with the precuneus gray matter volume, which was linked to the loss of brain structural integrity that occurs during the early stages of AD (Schaeverbeke et al., 2019). We found the volume of left precuneus was reduced in AD patients than aMCI patients or HC, while no significant difference was shown between aMCI and HC participants. Furthermore, the volume of left precuneus region displayed a significant correlation with either the MMSE score or MoCA score. The reduced volume of left precuneus region may be used as a marker for aMCI to AD. However, in the present study, we only found the proof of the correlation of the left precuneus region with AD progression. Whether the right precuneus region also involves in it, or only the left precuneus is specific for AD progression, needs more investigation. Besides, future research concentrating on the underlying molecular process will be required.

Additionally, we found that the AD group had more severe inferior parietal atrophies than the other two groups after completing SBM on all sMRIs. Figure 2 showed the AD patients had thinner left/right inferior parietal region, compared to the aMCI group and the HC group, and the thickness of either side of inferior parietal region displayed a significant correlation with either the MMSE score or MoCA score. This mean, with the more cognitive ability decreased, the thinner left/right inferior parietal became, suggesting the thickness of left/right inferior parietal region might correlate with the cognitive ability. The inferior parietal is situated behind the postcentral sulcus and below the horizontal part of the intraparietal sulcus (Torrey, 2007; Yang et al., 2023). Earlier research using functional MRI suggested that the inferior parietal was involved in an extensive spectrum of higher cognitive tasks (Desmurget and Sirigu, 2012; Igelstrom and Graziano, 2017). Similar to this, in our current investigation, participants in the AD group with the lowest MMSE and MoCA scores had inferior parietal regions where they differed significantly from other groups. Additionally, inferior parietal was a crucial substrate for a variety of mental activities, including fundamental language and attention. According to SBM analysis, we found that the AD group’s inferior parietal thickness differed significantly from that of the aMCI and HC groups in this study. The quantity or density of cells in a cortical column affects the thickness of the cortex. Our observed alterations in inferior parietal area thickness were strongly associated with AD’s typical clinical abnormalities. Furthermore, the inferior parietal area thickness displayed a significant correlation with either the MMSE score or MoCA score. The reduced thickness of inferior parietal area may be used as a marker for aMCI to AD. However, more research is required on this issue, and the thickness changes in the left/right inferior parietal in different brain status, such as language, attention, even diseases such as AD, should be compared and monitored.

Our current study still has some limitation, which is inevitable. Based on the standard aforementioned description, which is a cross-section reflecting each participant’s unique situation, our collected individuals were classified into three groups. Therefore, the only conclusion we can draw is that the significant difference among AD, aMCI, and HC found through SBM and VBM analyses may 1 day serve as a biomarker for the diagnosis of AD. According to our current findings, we will conduct cohort research to track the changes in the discovered brain regions as AD progresses as well as animal trials to support the brain regions’ predicted predictive value. Meanwhile, further research is still required to understand the underlying mechanisms by which these brain regions contribute to AD. We anticipate that our research will add a fresh perspective to the early identification and treatment of AD. Additionally, we will collect data on more patients while we continue conducting our study.

In conclusion, we collected sMRI data from participants in the AD, aMCI, and HC groups and performed VBM and SBM analyses. The left precuneus and both sides of the inferior parietal region were found to change in AD patients and might be new potential diagnostic biomarkers for AD progression.

## Data Availability

The original contributions presented in the study are included in the article/supplementary material, further inquiries can be directed to the corresponding authors.

## Footnotes

^1^http://www.alivelearn.net/xjview/

## References

[ref1] Bailly M. Destrieux C. Hommet C. Mondon K. Cottier J. P. Beaufils E.. (2015). Precuneus and cingulate cortex atrophy and Hypometabolism in patients with Alzheimer's disease and mild cognitive impairment: MRI and (18) F-FDG PET quantitative analysis using FreeSurfer. Biomed. Res. Int. 2015:583931. doi: 10.1155/2015/583931, PMID: 26346648 PMC4539420

[ref2] Balthazar M. L. Yasuda C. L. Pereira F. R. Bergo F. P. Cendes F. Damasceno B. P. (2010). Coordinated and circumlocutory semantic naming errors are related to anterolateral temporal lobes in mild AD, amnestic mild cognitive impairment, and normal aging. J. Int. Neuropsychol. Soc. 16, 1099–1107. doi: 10.1017/S1355617710000998 20887649

[ref3] Bayram E. Caldwell J. Z. K. Banks S. J. (2018). Current understanding of magnetic resonance imaging biomarkers and memory in Alzheimer's disease. Alzheimers Dement. 4, 395–413. doi: 10.1016/j.trci.2018.04.007, PMID: 30229130 PMC6140335

[ref4] Biffen S. C. Warton C. M. R. Dodge N. C. Molteno C. D. Jacobson J. L. Jacobson S. W.. (2020). Validity of automated FreeSurfer segmentation compared to manual tracing in detecting prenatal alcohol exposure-related subcortical and corpus callosal alterations in 9- to 11-year-old children. Neuroimage Clin. 28:102368. doi: 10.1016/j.nicl.2020.102368, PMID: 32791491 PMC7424233

[ref5] Breijyeh Z. Karaman R. (2020). Comprehensive review on Alzheimer's disease: causes and treatment. Molecules 25:5789. doi: 10.3390/molecules25245789, PMID: 33302541 PMC7764106

[ref6] Brier M. R. Gordon B. Friedrichsen K. McCarthy J. Stern A. Christensen J.. (2016). Tau and Aβ imaging, CSF measures, and cognition in Alzheimer's disease. Sci. Transl. Med. 8:338ra66. doi: 10.1126/scitranslmed.aaf2362, PMID: 27169802 PMC5267531

[ref7] Brown E. M. Pierce M. E. Clark D. C. Fischl B. R. Iglesias J. E. Milberg W. P.. (2020). Test-retest reliability of FreeSurfer automated hippocampal subfield segmentation within and across scanners. NeuroImage 210:116563. doi: 10.1016/j.neuroimage.2020.116563, PMID: 31972281

[ref8] Caunca M. R. Siedlecki K. Cheung Y. K. Alperin N. Lee S. H. Elkind M. S. V.. (2020). Cholinergic white matter lesions, AD-signature cortical thickness, and change in cognition: the northern Manhattan study. J. Gerontol. A Biol. Sci. Med. Sci. 75, 1508–1515. doi: 10.1093/gerona/glz279, PMID: 31944231 PMC7457185

[ref9] Chen W. Li S. Ma Y. Lv S. Wu F. Du J.. (2021). A simple nomogram prediction model to identify relatively young patients with mild cognitive impairment who may progress to Alzheimer's disease. J. Clin. Neurosci. 91, 62–68. doi: 10.1016/j.jocn.2021.06.026, PMID: 34373060

[ref10] Cooray R. Gupta V. Suphioglu C. (2020). Current aspects of the endocannabinoid system and targeted THC and CBD Phytocannabinoids as potential therapeutics for Parkinson's and Alzheimer's diseases: a review. Mol. Neurobiol. 57, 4878–4890. doi: 10.1007/s12035-020-02054-6, PMID: 32813239 PMC7515854

[ref11] Cui L. Yin H. Lyu S. Shen Q. Wang Y. Li X.. (2019). Tai chi Chuan vs general aerobic exercise in brain plasticity: a multimodal MRI study. Sci. Rep. 9:17264. doi: 10.1038/s41598-019-53731-z, PMID: 31754170 PMC6872722

[ref12] de Oliveira T. R. Erbereli C. R. Manzine P. R. Magalhaes T. N. C. Balthazar M. L. F. Cominetti M. R.. (2020). Early diagnosis of Alzheimer's disease in blood using a disposable electrochemical microfluidic platform. ACS Sens. 5, 1010–1019. doi: 10.1021/acssensors.9b02463, PMID: 32207606

[ref13] De Wit L. Piai V. Thangwaritorn P. Johnson B. O'Shea D. Amofa P. Sr.. (2022). Repetition priming in individuals with amnestic mild cognitive impairment and Alzheimer's dementia: a systematic review and Meta-analysis. Neuropsychol. Rev. 32, 228–246. doi: 10.1007/s11065-021-09504-5, PMID: 33895980 PMC9090892

[ref14] Desmurget M. Sirigu A. (2012). Conscious motor intention emerges in the inferior parietal lobule. Curr. Opin. Neurobiol. 22, 1004–1011. doi: 10.1016/j.conb.2012.06.006, PMID: 22939569

[ref15] Dugu M. Neugroschl J. Sewell M. Marin D. (2003). Review of dementia. Mt Sinai J. Med. 70, 45–53, PMID: 12516009

[ref16] Feczko A. (2014). Dementia in the incarcerated elderly adult: innovative solutions to promote quality care. J. Am. Assoc. Nurse Pract. 26, 640–648. doi: 10.1002/2327-6924.12189 25384367

[ref17] Feng J. Zhang S. W. Chen L. (2022). Extracting ROI-based Contourlet subband energy feature from the sMRI image for Alzheimer's disease classification. IEEE/ACM Trans. Comput. Biol. Bioinform. 19, 1627–1639. doi: 10.1109/TCBB.2021.3051177, PMID: 33434134

[ref18] Finney G. R. Minagar A. Heilman K. M. (2016). Assessment of mental status. Neurol. Clin. 34, 1–16. doi: 10.1016/j.ncl.2015.08.001, PMID: 26613992

[ref19] Giannouli V. Tsolaki M. (2019). Are left angular gyrus and amygdala volumes important for financial capacity in mild cognitive impairment? Hell. J. Nucl. Med. 22, 160–164, PMID: 30877733

[ref20] Giannouli V. Tsolaki M. (2023). Brain volumes and metacognitive deficits in knowledge of self, task and strategies in mathematics: a preliminary pilot one-year longitudinal study in aMCI patients compared to healthy controls. Diagnostics 13:680. doi: 10.3390/diagnostics13040680, PMID: 36832169 PMC9955851

[ref21] Giridharan V. V. Barichello De Quevedo C. E. Petronilho F. (2022). Microbiota-gut-brain axis in the Alzheimer's disease pathology - an overview. Neurosci. Res. 181, 17–21. doi: 10.1016/j.neures.2022.05.003, PMID: 35577241

[ref22] Igelstrom K. M. Graziano M. S. A. (2017). The inferior parietal lobule and temporoparietal junction: a network perspective. Neuropsychologia 105, 70–83. doi: 10.1016/j.neuropsychologia.2017.01.001, PMID: 28057458

[ref23] Jack C. R. Jr. Bennett D. A. Blennow K. Carrillo M. C. Dunn B. Haeberlein S. B.. (2018). NIA-AA research framework: toward a biological definition of Alzheimer's disease. Alzheimers Dement. 14, 535–562. doi: 10.1016/j.jalz.2018.02.018, PMID: 29653606 PMC5958625

[ref24] Jagust W. (2008). Is amnestic mild cognitive impairment always AD? Neurology 70, 502–503. doi: 10.1212/01.wnl.0000299190.17488.b3, PMID: 18268243

[ref25] Jia L. Qiu Q. Zhang H. Chu L. Du Y. Zhang J.. (2019). Concordance between the assessment of Abeta42, T-tau, and P-T181-tau in peripheral blood neuronal-derived exosomes and cerebrospinal fluid. Alzheimers Dement. 15, 1071–1080. doi: 10.1016/j.jalz.2019.05.002, PMID: 31422798

[ref26] Khatri U. Kwon G. R. (2022). Alzheimer's disease diagnosis and biomarker analysis using resting-state functional MRI functional brain network with multi-measures features and hippocampal subfield and amygdala volume of structural MRI. Front. Aging Neurosci. 14:818871. doi: 10.3389/fnagi.2022.818871, PMID: 35707703 PMC9190953

[ref27] Koch G. Bonni S. Pellicciari M. C. Casula E. P. Mancini M. Esposito R.. (2018). Transcranial magnetic stimulation of the precuneus enhances memory and neural activity in prodromal Alzheimer's disease. NeuroImage 169, 302–311. doi: 10.1016/j.neuroimage.2017.12.048, PMID: 29277405

[ref28] Kowalska J. Mazurek J. Kubasik N. Rymaszewska J. (2019). Effectiveness of physiotherapy in elderly patients with dementia: a prospective, comparative analysis. Disabil. Rehabil. 41, 815–819. doi: 10.1080/09638288.2017.1410859, PMID: 29189083

[ref29] Li Y. Gu B. J. (2023). Non-human primate models of Alzheimer’s disease. J. Explor. Res. Pharm. 8, 200–222. doi: 10.14218/jerp.2023.00006

[ref30] Liu Y. Zhuang D. Wang J. Huang H. Li R. Wu C.. (2022). Recent advances in small molecular near-infrared fluorescence probes for a targeted diagnosis of the Alzheimer disease. Analyst 147, 4701–4723. doi: 10.1039/D2AN01327D, PMID: 36190126

[ref31] Mahaman Y. A. R. Embaye K. S. Huang F. Li L. Zhu F. Wang J. Z.. (2022). Biomarkers used in Alzheimer's disease diagnosis, treatment, and prevention. Ageing Res. Rev. 74:101544. doi: 10.1016/j.arr.2021.101544, PMID: 34933129

[ref32] Marcucci V. Kleiman J. (2021). Biomarkers and their implications in Alzheimer’s disease: a literature review. Explor. Res. Hypoth. Med. 6, 164–176. doi: 10.14218/erhm.2021.00016

[ref33] Mlinac M. E. Feng M. C. (2016). Assessment of activities of daily living, self-care, and Independence. Arch. Clin. Neuropsychol. 31, 506–516. doi: 10.1093/arclin/acw049, PMID: 27475282

[ref34] Rajmohan R. Reddy P. H. (2017). Amyloid-Beta and Phosphorylated tau accumulations cause abnormalities at synapses of Alzheimer's disease neurons. J. Alzheimers Dis. 57, 975–999. doi: 10.3233/JAD-160612, PMID: 27567878 PMC5793225

[ref35] Rechberger S. Li Y. Kopetzky S. J. Butz-Ostendorf M. (2022). Automated high-definition MRI processing routine robustly detects longitudinal morphometry changes in Alzheimer's disease patients. Front. Aging Neurosci. 14:832828. doi: 10.3389/fnagi.2022.832828, PMID: 35747446 PMC9211026

[ref36] Regan P. McClean P. L. Smyth T. Doherty M. (2019). Early stage glycosylation biomarkers in Alzheimer's disease. Medicines 6:92. doi: 10.3390/medicines6030092, PMID: 31484367 PMC6789538

[ref37] Rosenblatt A. (2005). The art of managing dementia in the elderly. Cleve. Clin. J. Med. 72, S3–S13. doi: 10.3949/ccjm.72.Suppl_3.S3, PMID: 16265939

[ref38] Schaeverbeke J. Gille B. Adamczuk K. Vanderstichele H. Chassaing E. Bruffaerts R.. (2019). Cerebrospinal fluid levels of synaptic and neuronal integrity correlate with gray matter volume and amyloid load in the precuneus of cognitively intact older adults. J. Neurochem. 149, 139–157. doi: 10.1111/jnc.14680, PMID: 30720873

[ref39] Seijo-Martinez M. Cancela J. M. Ayán C. Varela S. Vila H. (2016). Influence of cognitive impairment on fall risk among elderly nursing home residents. Int. Psychogeriatr. 28, 1975–1987. doi: 10.1017/S1041610216001113, PMID: 27605458

[ref40] Sundermann E. E. Bondi M. W. Campbell L. M. Gouaux B. Moore R. C. Soontornniyomkij V.. (2021). Distinguishing amnestic mild cognitive impairment from HIV-associated neurocognitive disorders. J. Infect. Dis. 224, 435–442. doi: 10.1093/infdis/jiaa760, PMID: 33319235 PMC8328198

[ref41] Thomann A. K. Schmitgen M. M. Kmuche D. Ebert M. P. Thomann P. A. Szabo K.. (2021). Exploring joint patterns of brain structure and function in inflammatory bowel diseases using multimodal data fusion. Neurogastroenterol. Motil. 33:e14078. doi: 10.1111/nmo.14078, PMID: 33368950

[ref42] Tomoto T. Liu J. Tseng B. Y. Pasha E. P. Cardim D. Tarumi T.. (2021). One-year aerobic exercise reduced carotid arterial stiffness and increased cerebral blood flow in amnestic mild cognitive impairment. J. Alzheimers Dis. 80, 841–853. doi: 10.3233/JAD-201456, PMID: 33579857

[ref43] Torrey E. F. (2007). Schizophrenia and the inferior parietal lobule. Schizophr. Res. 97, 215–225. doi: 10.1016/j.schres.2007.08.023 17851044

[ref44] van Oostveen W. M. de Lange E. C. M. (2021). Imaging techniques in Alzheimer's disease: a review of applications in early diagnosis and longitudinal monitoring. Int. J. Mol. Sci. 22:2110. doi: 10.3390/ijms22042110, PMID: 33672696 PMC7924338

[ref45] Verhulst M. Glimmerveen A. B. van Heugten C. M. Helmich R. C. G. Hofmeijer J. (2023). MRI factors associated with cognitive functioning after acute onset brain injury: systematic review and meta-analysis. Neuroimage Clin. 38:103415. doi: 10.1016/j.nicl.2023.103415, PMID: 37119695 PMC10165272

[ref46] Wang H. Dey K. K. Chen P. C. Li Y. Niu M. Cho J. H.. (2020). Integrated analysis of ultra-deep proteomes in cortex, cerebrospinal fluid and serum reveals a mitochondrial signature in Alzheimer's disease. Mol. Neurodegener. 15:43. doi: 10.1186/s13024-020-00384-6, PMID: 32711556 PMC7382148

[ref47] Whitwell J. L. Shiung M. M. Przybelski S. A. Weigand S. D. Knopman D. S. Boeve B. F.. (2008). MRI patterns of atrophy associated with progression to AD in amnestic mild cognitive impairment. Neurology 70, 512–520. doi: 10.1212/01.wnl.0000280575.77437.a2, PMID: 17898323 PMC2734138

[ref48] Yang K. Du R. Yang Q. Zhao R. Fan F. Chen S.. (2023). Cortical thickness of the inferior parietal lobule as a potential predictor of relapse in men with alcohol dependence. Res. Sq. doi: 10.21203/rs.3.rs-2628081/v1, PMID: [Preprint]. 38078981

[ref49] ZhangX. X.TianY.WangZ. T.MaY. H.TanL.YuJ. T. (2021). The epidemiology of Alzheimer's disease modifiable risk factors and prevention. J. Prev. Alzheimers Dis. 8, 1–9. doi: 10.14283/jpad.2021.1534101789

[ref50] Zhao R. Wang P. Liu L. Zhang F. Hu P. Wen J.. (2023). Whole-brain structure-function coupling abnormalities in mild cognitive impairment: a study combining amplitude of low-frequency fluctuations and voxel-based morphometry. Front. Neurosci. 17:1236221. doi: 10.3389/fnins.2023.1236221, PMID: 37583417 PMC10424122

[ref51] Zhu Z. Huang T. Zhen Z. Wang B. Wu X. Li S. (2023). From sMRI to task-fMRI: a unified geometric deep learning framework for cross-modal brain anatomo-functional mapping. Med. Image Anal. 83:102681. doi: 10.1016/j.media.2022.102681, PMID: 36459804

